# Organotypic model of the gingiva for studying bacterial and viral pathogens implicated in periodontitis

**DOI:** 10.1080/20002297.2023.2292382

**Published:** 2023-12-12

**Authors:** Anna Golda, Anna Gasiorek, Ewelina Dobosz, Zuzanna Oruba, Richard J. Lamont, Jan Potempa, Joanna Koziel

**Affiliations:** a Department of Microbiology, Faculty of Biochemistry, Biophysics and Biotechnology of Jagiellonian University, Krakow, Poland; b Department of Periodontology, Preventive Dentistry and Oral Pathology, Jagiellonian University Medical College, Faculty of Medicine, Krakow, Poland; c Department of Oral Immunology and Infectious Diseases, University of Louisville School of Dentistry, University of Louisville, Louisville, KY, USA

**Keywords:** Organotypic, gingival model, *Porphyromonas gingivalis*, herpes simplex virus-1, infection, gingival tissue, persistence, biofilm

## Abstract

**Background:**

Three-dimensional (3D) tissue models bridge the gap between conventional two-dimensional cell cultures and animal models. The aim of this study was to develop an organotypic 3D gingival (OTG) model to provide a tool to investigate bacterial and viral pathogens in periodontitis.

**Methods:**

The OTG model composed of gingival fibroblasts (GFs) and telomerase-immortalized gingival keratinocytes (TIGKs) was constructed and applied to study infections by *Porphyromonas gingivalis* and herpes simplex virus 1 (HSV-1). Immunohistochemical staining, confocal microscopy, qPCR, titration techniques, and colony-forming unit counts were applied to interrogate epithelial markers expression, monitor *P. gingivalis* and HSV-1 presence, and evaluate the immune response along with the efficiency of antimicrobial drugs.

**Results:**

The OTG model resembled the morphology of the human gingiva. During infection, both pathogens penetrated deep into the tissue and persisted for a few days with *P. gingivalis* also forming a biofilm on the cell surface. The infection triggered the expression of inflammatory mediators in cells and both pathogens were efficiently eliminated by specific antimicrobials.

**Conclusions:**

Presented OTG model constitutes a simple and convenient tool to study the interaction between bacterial and viral pathogens within the gingival tissue, including penetration, persistence and biofilm formation. It is also suitable to examine the efficiency of antimicrobial drugs.

## Introduction

The use of three-dimensional (3D) tissue models is gaining popularity to bridge the gap between conventional two-dimensional (2D) cell cultures and animal models. These models include organotypic models of gingival tissue used in oral irritation and cytotoxicity screening of oral care products (toothpastes and mouthwashes) [1], assessment of responses to tobacco products (e-cigarettes) [2], and drug testing. Organotypic cultures are also urgently needed to study highly prevalent gingival infections, and their use in this field is constantly evolving. Periodontitis is an inflammatory disease of the marginal periodontium with bacterial etiology that affects approximately 30% of the human population [3]. The infectious agents of periodontitis include *Porphyromonas gingivalis*, a Gram-negative anaerobe which is found in majority of subgingival plaque samples from patients with chronic periodontitis [4]. *P. gingivalis* significantly modulates the response of gingival tissue, favoring the proliferation of other bacterial pathobionts, which collectively induce chronic inflammation and promote tissue destruction. Therefore, *P. gingivalis* was recently defined as a keystone pathogen in periodontitis development [5,6]. However, an increased number of clinical reports also indicate the significant role of different viruses that interact with the subgingival dysbiotic biofilm [7] and lead to impairment of the host’s immune response. These viruses include herpes simplex virus 1 (HSV-1), Epstein-Barr Virus (EBV) and Human Cytomegalovirus (HCMV) whose occurrence is associated with bacterial species related to periodontitis (e.g. *P. gingivalis*, *Tannerella forsythia*, and *Prevotella intermedia*) [8–13]. Therefore, it is crucial to understand the interaction between periodontal pathogens and the epithelium in detail, to prevent and treat periodontitis. The introduction of organotypic cultures into routine laboratory practice will provide a valuable tool to investigate the molecular basis of this disease.

There is a large variety of organotypic models depending on their intended use, and thus they vary in terms of the scaffold, type of cells, and culture medium. Informed selection of these factors ensures the design of an optimal model dedicated to specific research questions. Moreover, the model should recapitulate gingival tissue to the extent possible. The most crucial part of an organotypic gingival model is the use of suitable epithelial cells. These can be primary gingival epithelial cells (GECs) [14,15] or well-established cell lines immortalized with HPV16 E6/E7 [16–19] or SV-40 [20] genes. Both approaches have limitations because GECs undergo senescence, while immortalized cell lines can be affected by chromosomal changes. The introduction of telomerase-immortalized gingival keratinocytes (TIGKs) opened new possibilities because these cells have similar characteristics as parental primary GECs [21]. TIGKs have been applied in an organotypic model with success [22] but not for infection studies.

The virulence of *P. gingivalis* was studied in many 2D cell cultures but recently 3D models were developed and used intensively to investigate different aspects of pathogenic interactions of pathogens with the host [20,23–25]. Nevertheless, there are limited studies showing viral infection in an organotypic gingival model, with none using HSV-1 to the best of our knowledge [26–28]. Therefore, our objective was to optimize an organotypic gingival model composed of donor-independent and easily accessible human cell lines, which will provide a valuable tool to investigate the interactions of bacterial and/or viral pathogens with the host. The presented OTG model is composed of TIGKs and immortalized human gingival fibroblasts (GFs), which form an epithelial layer that resembles the regular stratified gingival epithelium. We further assessed whether this model can be used to study infection with different types of oral pathogens, including bacteria and viruses. We adjusted the model to study short- and long-term infections with *P. gingivalis* and showed that it can be used to differentiate the response of gingival tissue to bacterial strains with different virulence characteristics. In addition, we assessed whether this model can be used as a tool to study viral infections using HSV-1.

## Materials and methods

### Reagents

Fetal bovine serum (FBS), Phosphate buffered saline (PBS), Dulbecco’s modified eagle medium (DMEM), penicillin, and streptomycin were purchased from Gibco. Bovine collagen type I, hydrocortisone, o-phosphatrolysine (OPS), adenine, progesterone, triiodothyronine, ITES, Gentamicin, brain heart infusion (BHI), and hematoxylin-eosin (H&E) were from Sigma-Aldrich. Newborn calf serum, HAM’s F12 medium, L-glutamine, TaqMan^TM^ Fast Advanced Master Mix, TaqMan^TM^ Microbe Detection assay against HSV-1, InsTAclone PCR cloning kit, pTZ57R/T plasmid, GeneJet Plasmid Miniprep Kit, poly-L-lysine coated glass slides, ProLong Antifade Mounting Medium with DAPI were obtained from ThermoFisher Scientific.

### Cell lines

Telomerase-immortalized gingival keratinocytes (TIGKs) were generated from primary gingival epithelial cells (GECs) [21]. Cells were cultivated in KBM-Gold keratinocyte basal medium supplemented with Single Quots (Lonza). Immortalized Human Gingival Fibroblasts-hTERT (T0026, Applied Biological Materials) were cultivated on collagen-coated bottles in PriGrow III medium (Applied Biological Materials) supplemented with 10% heat-inactivated fetal bovine serum (FBS, Life Technologies). Vero E6 cells (ATCC CRL-1586) were routinely maintained in DMEM supplemented with 3% heat-inactivated FBS, penicillin (100 U/mL), and streptomycin (100 μg/mL). Cell cultures were maintained in the atmosphere containing 5% CO_2_ at 37°C, and when they reached 80–90% confluency, cells were seeded for experiments.

### Gingival tissue specimens

Gingival tissue specimens were collected at the Department of Periodontology Dental University Clinic, Kraków, Poland. The donors were systemically healthy, without a relevant history of medication intake. Based on the full-mouth periodontal charting, the patients were categorized as ‘gingival health on an intact periodontium’ according to the Classification of Periodontal and Peri-Implant Diseases and Conditions [29]. The tissue samples were harvested in the course of crown-lengthening surgical procedures due to aesthetic indications. This study was approved by and carried out in accordance with the recommendations of the Bioethical Committee of the Jagiellonian University in Kraków, Poland (permit numbers 1072.6120.176.2020). All subjects gave written informed consent in accordance with the Declaration of Helsinki.

### Construction of a 3D gingiva model

Protocol was modified from that described previously [30]. To prepare 3D cultures, 150,000 gingival fibroblasts suspended in 10% FBS in DMEM were mixed with 2.4 ml of ice-cold Matrigel® (Corning) (final concentration 7 mg/mL) and placed in the middle of 12-well cell culture inserts (Greiner). The cells were incubated for 2 h at 37°C, 5% CO_2_ without culture medium, to allow the matrix to solidify. After this, 0.5 ml of culture medium (10% FBS, 100 U/mL penicillin, and 100 ug/mL streptomycin in DMEM) was added to the top of the inserts, and 1.5 ml of medium to the bottom of the inserts. The next day, with the use of a mini scalpel blade, the circumference of the matrix was outlined, and the medium was changed to the fresh one. Three days later, 1 x 10^6 TIGK cells suspended in EPM1 (epidermalization medium 1 - Table 1) were added to the top of the insert. The culture medium was changed every 2 days. On the ninth day after the start of the procedure, the old culture medium was removed and EPM2 (epidermalization medium 2 - Table 1) was added to the bottom of the insert. The epithelial layer on the top was exposed to the air, which promotes epithelial differentiation and then stratification. About 8–10 days after air-liquid interphase, 3D cultures of gingiva were used for further experiments.

**Table 1.** The composition of EPM1 and EPM2.

Components	EPM1	EPM2
DMEM:Ham’s F12	3:1	1:1
L-Glutamine	4mM	4mM
Hydrocortisone	0.15 μM	0.15 μM
ITES Supplement		
Isnulin	10 μg/mL	10 μg/mL
Transferrin	10 μg/mL	10 μg/mL
Ethanolamine	10 μg/mL	10 μg/mL
Selenium	10 μg/mL	10 μg/mL
*O*-Phosporylethanolamine	0.01 mM	0.01 mM
Adenine	0.18 mM	0.18 mM
Progesterone	4 pM	-
Triiodothyronine	20 pM	20 pM
Newborn Calf Serum	0.1%	2%
Gentamicin sulfate	0.05 mg/mL	0.05 mg/mL

### Bacterial infection

*P. gingivalis* ATCC 33277 was routinely grown in BHI medium supplemented with 10 μg/L hemin (BioShop) and 0.5 μg/mL menadione (ICN Biomedicals), the W83 strain was grown in TSB medium supplemented with yeast extract, 5 μg/L hemin, 50 μg/ml l-cysteine (BioShop) and 0.5 μg/mL menadione. All strains were cultivated in anaerobic conditions (90% N_2_, 5% H_2_, and 5% CO_2_) at 37°C. After overnight culture bacteria were centrifuged (5,000 rpm, 10 min), then the pellet was washed three times and resuspended in PBS at a final optical density OD_600_ = 1. To induce infection of the 3D model, a bacterial suspension was added on the apical side of the insert − 4 x 10^7 of *P*. *gingivalis* cells in the case of a single infection, every two days (1d, 3d, 5d, 7d), or 2 x 10^7 of cells in the case of multiple infections. After 2 h, the remaining bacterial suspension was gently removed from the insert. When necessary, metronidazole (100 μg/mL) was added for 2 h to eliminate the remaining bacteria. The infection was continued at designated times, and the material was harvested for further analysis.

### Quantification of bacterial DNA by quantitative PCR

To isolate total DNA from the OTG model, keratinocytes were removed from the matrix using a scalpel blade. DNA was isolated using a commercially available kit according to the manufacturer’s instructions (DNeasy® Blood & Tissue, Qiagen). To generate a standard curve, different dilutions of bacterial culture were prepared, and total DNA was isolated. Next, 1 μL of a sample was mixed with 10 μM specific primers for *P. gingivalis* 16s rRNA (forward 5’AGGCAGCTTGCCATACTGCG 3’ and reverse 5’ACTGTTAGCAACTACCGATGT 3’) and GoTaq qPCR Master Mix (Promega). The total number of bacteria was calculated from the standard curve as previously described [31]. To confirm the specificity of the product, we performed a melt curve analysis.

### Virus preparation and titration

HSV-1 stocks were generated by infecting Vero E6 cells. Cells were lysed at 48 h p.i. by two freeze-thaw cycles. The fluid containing the virus was aliquoted and stored at −80°C. A control Vero E6 cell lysate from mock-infected cells was prepared in the same way as the virus stock. The virus yield, in stock or collected samples, was evaluated by virus titration on fully confluent Vero E6 cells in 96-well plates, according to the method of Reed & Muench [32]. The plates were incubated at 37°C for 48 h, the occurrence of a cytopathic effect was scored using an inverted microscope, and TCID_50_ (50% tissue culture infection dose) was calculated.

### Viral infection

Briefly, the apical surface of OTG cells was washed three times with PBS and then inoculated with 100 μL of viral stock (TCID_50_ 400 or 2000/ml). Following incubation for 2 h at 37°C, the unbound HSV-1 was removed by washing 3x with PBS for 10 min at 37°C, and the OTG cultures were maintained at an air-liquid interface for the rest of the experiment. To determine HSV-1 replication kinetics, 120 μL of PBS was applied to the apical surface of the OTG, and harvested for DNA isolation after 10 min of incubation at 37°C.

### Virus detection by qPCR

Viral DNA was isolated from the apical washes of inserts using the Viral DNA/RNA Isolation Kit (A&A Biotechnology). The virus yield was assessed by quantitative real-time PCR (qPCR). The reaction was carried out in a CFX96 Touch Real-Time PCR Detection System (Bio-Rad), in a 10 μL reaction mixture consisting of TaqMan^TM^ Fast Advanced Master Mix, TaqMan^TM^ Microbe Detection assay against HSV-1 (Vi04230116_s1) and 1 μL of viral DNA. The temperature profile was 20 s at 95°C, followed by 40 cycles of 3 s at 95°C and 20 s at 60°C. DNA quantification standards were prepared. In summary, a fragment of viral DNA was amplified using the primers listed above and cloned into the pTZ57R/T plasmid using the InsTAclone PCR cloning kit. The plasmid was propagated in *E. coli*, purified with the GeneJet Plasmid Miniprep Kit, and digested with the EcoR1 restriction enzyme. The number of copies per ml was estimated after the concentration of linearized DNA was assessed spectrophotometrically.

### Histological staining of tissues

Briefly, cell inserts were fixed in 10% formaldehyde at 4°C for 1 h. Then, the inserts along with the membrane were excised with a scalpel, washed three times in PBS, and dehydrated with graded series of ethanol (from 50% to 100%) and xylene. After embedding in paraffin, 3D cultures were cut into 5 μm sections and mounted on poly-L-lysine coated glass slides, then sections were dewaxed in xylene, rehydrated with a series of gradually increasing ethanol (from 100% to 50%) and stained with H&E solution. For immunohistochemical analysis, slides were heated for 20 min in sodium citrate buffer (10 mM sodium citrate and 0.05% Tween 20 [pH 6.0]), incubated for 1 h at room temperature in blocking buffer (5% normal goat serum, 0.1% saponin in PBS) and stained with a set of primary antibodies: anti-keratin 10 (Abcam), -keratin 14 (Abcam), -PCNA (Dako), -vimentin (Abcam), -HSV (Abcam), -*P. gingivalis* (Sigma-Aldrich) (1:100, 5% normal goat serum, 0.1% saponin in PBS). After several washes slides were incubated for 45 min at room temperature with secondary antibodies: goat anti-rabbit or goat anti-mouse conjugated with Alexa Fluor 488 (1:500; Cell Signaling). Finally, cell nuclei were stained with ProLong Antifade Mounting Medium with DAPI. Slides were visualized with EVOS FL Cell Imaging System (Thermo Fisher Scientific). The quantification of the fluorescence signal was carried out in ImageJ software. Briefly, the mean fluorescence of the green signal was measured in the rectangle area (188 × 83 pixels) in the images without any graphical corrections. From 11 to 18 the field of view was captured and presented as mean ± standard deviation (SD).

### Confocal scanner microscopy

Cell inserts after fixation and incubation with blocking buffer were stained with primary antibodies (1:100; rabbit anti-*P. gingivalis* and anti-HSV) for 1 h at room temperature. The inserts were then incubated with secondary antibodies (1:500; goat anti-rabbit conjugated with Alexa Fluor 488) and with Alexa Fluor 647 Phalloidin. After several washes with PBS, the entire gingiva model was excised with a scalpel and gently placed on a glass slide. Images were captured with a confocal laser scanning microscope (LSM 880; Zeiss) and visualized in ZEISS ZEN.

### Analysis of genes expression

Briefly, total cellular RNA from keratinocytes was isolated using a total RNA Minikit (A&A), transcribed to cDNA using a high-capacity cDNA reverse transcription kit (Applied Biosystems) and quantitative reverse transcription-PCR (qPCR) was performed. The PCR was carried in a mixture containing 40 ng of cDNA, 10 mM forward and reverse primers and 1 × GoTaq qPCR master mix (Promega). The primer sequences with product lengths and the GeneBank accession number are listed in Table 2. The initial denaturation step were performed at 95°C for 5 min; then reactions were carried out for 40 cycles (95°C, 30 s; 56°C, 30 s; 72°C, 45 s), followed by a final elongation step at 72°C for 10 min. qPCR was carried out for up to 40 cycles, the cycle threshold values were quantified and analyzed with 2^−ΔΔ^*^C^*_T_ method [33]. To confirm the specificity of the products, a melt curve analysis was performed.

**Table 2.** The primer sequences with base pair lengths and the GeneBank accession number.

Gene	GenBank Accession	Sequence	Product length
*EF-2 F*	NM_001961	5’- GACATCACCAAGGGTGTGCAG-3’	215 bp
*EF-2 R*		5’- TTCAGCACACTGGCATAGAGGC-3’	
*IL-6 F*	NM_000600	5’- CATCTTTGGAAGGTTCAGGTTGT-3’	91 bp
*IL-6 R*		5’- AGCCCTGAGAAAGGAGACATGTA-3’	
*IL-8 F*	NM_000584	5’-ATGACTTCCAAGCTGGCCGTGGCT-3	292 bp
*IL-8 R*		5’-TCTCAGCCCTCTTCAAAAACTTCT-3’	
*Ki67 F*	NM_002417	5’- TGACCCTGATGAGAAAGCTCAA-3’	141 bp
*Ki67 R*		5’- CCCTGAGCAACACTGTCTTTT-3’	
*IFN-β1 F*	NM_002176	5’- AAACTCATGAGCAGTCTGCA-3’	168 bp
*IFN-β1 R*		5’- AGGAGATCTTCAGTTTCGGAGG-3’	
*IFN-κ F*	NM_020124	5’- GCCCCAAGAGTTTCTGCAATAC-3’	79 bp
*IFN-κ R*		5’- GGCCTGTAGGGACATTTCATAGA-3’	
*IFN-λ F*	NM_172140	5’- GAAGCAGTTGCGATTTAGCC-3’	170 bp
*IFN-λ R*		5’- GAAGCTCGCTAGCTCCTGTG-3’	

### Statistical analysis

Data were expressed as means ± SD or as means ± SEM. Statistical significance was determined using Student *t*-test, one-way ANOVA, and/or two-way ANOVA for direct comparisons between single groups. A *p*-value <0.05 was considered statistically significant. We used GraphPad Prism 9.0 software for the analyses.

## Results

### Morphological characteristic of the 3D gingival model

**Figure 1.**
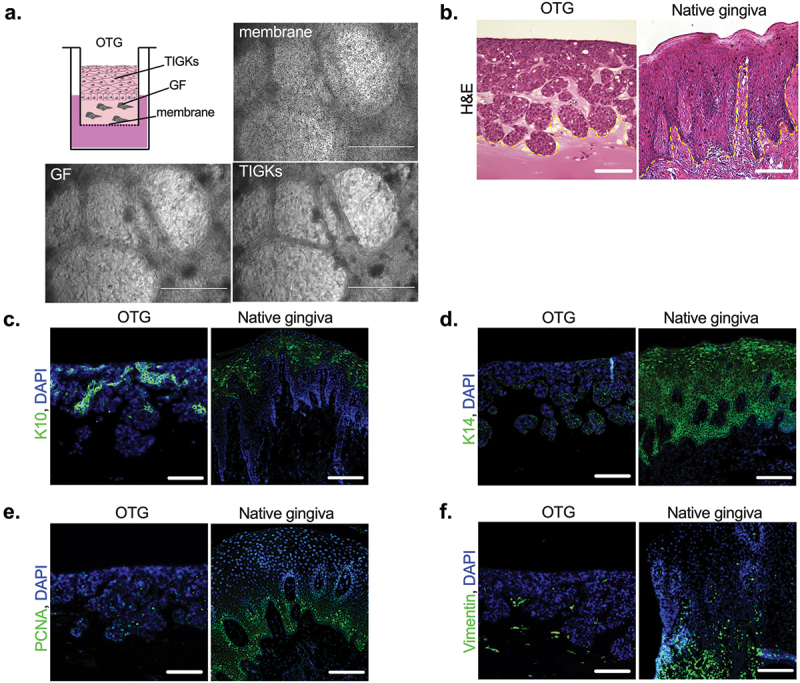
Morphological characteristic of the 3D gingival model. The 3D gingival model constructed from established cell lines closely represents the native gingival tissue architecture. The OTG model was constructed from GFs embedded in Matrigel® and TIGKs for the epithelial cell layer, as described in the material and Methods section. (a) Schematic drawing of the OTG model and representative microscopic images of the membrane, GFs, and TIGKs captured at the center of the culture insert by light microscopy. (b – f) Paraffin-embedded tissue sections were stained with H&E (b) To visualize morphology the multilayered (stratified) structure of the epithelium is distinguished from the connective tissue layer and fibroblasts (dashed line) located below. Immunohistochemically stained tissue processed with antibodies against epithelial (c, K10 and d, K14; green), proliferation (e, PCNA; green), and fibroblast (f, vimentin; green) biomarkers. Nuclei were stained with DAPI (blue). Scale bar in (a): 1000 μm; scale bar in (b – f): 100 μm.

Useful tools for the analysis of pathogen-host interactions include organotypic models that better reflect the infection process compared to 2D culture. Therefore, we aimed to design an organotypic 3D gingival (OTG) model that reflects the gingival tissue and is easily manipulated under laboratory conditions. The OTG was assembled from a connective tissue layer composed of Matrigel® matrix, GFs, and TIGKs (Figure 1a). We found that our organotypic 3D construct resembled the native gingiva, with the multilayered (stratified) structure of the epithelium distinguished (dashed line) from the connective tissue layer located below, where fibroblasts were embedded in the collagen hydrogel (Figure 1b). The expression pattern of the keratins K10 and K14 was similar to that in the native gingiva, with domination of K10 in the apical layers and wide distribution of K14 (Figure 1c,d). Proliferating cell nuclear antigen (PCNA) expression was confined to the basal layer, indicating the proliferation of keratinocytes (Figure 1e). Vimentin was used as a marker of fibroblasts that reside in connective tissue (Figure 1f). The data obtained show that the OTG model closely mimics the native gingiva.

### Monitoring the development of bacterial infection in the OTG model

**Figure 2.**
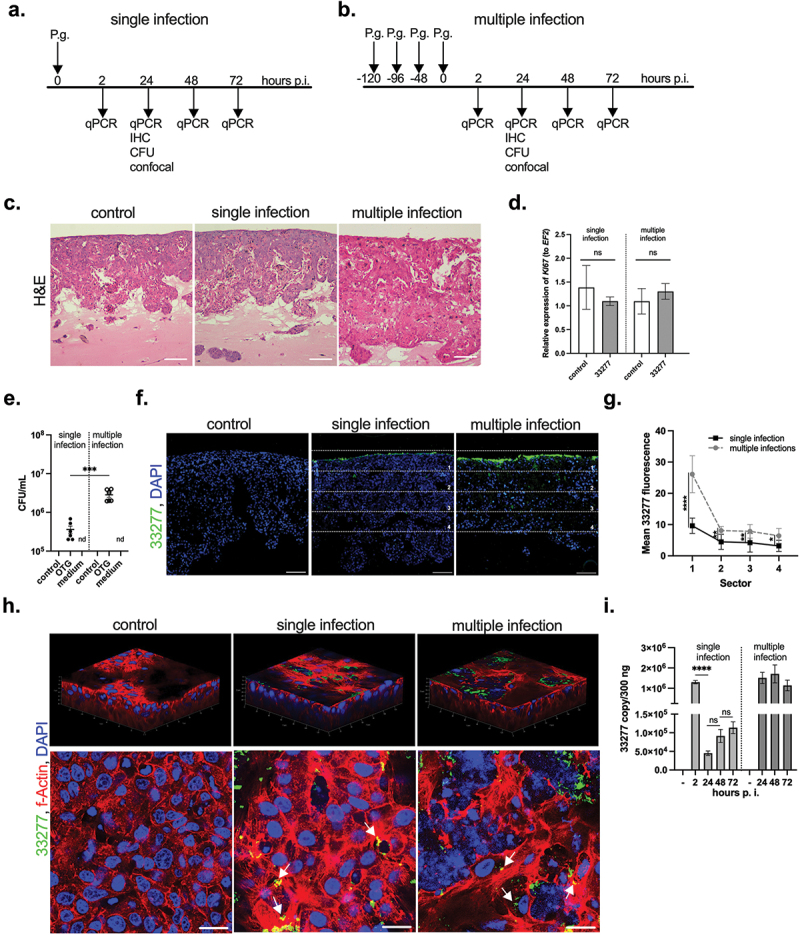
*P. gingivalis* infection of the 3D gingival model. The OTG model was used to investigate infection with *P. gingivalis*. Two models of infection were applied: single (a) and multiple (b) inoculations. The latter is thought to reflect chronic exposure to bacteria. (c) The morphology of the OTG model infected with 33277 was evaluated using H&E staining; scale bar, 100 μm. (d) Infection with 33277 has no influence on *Ki67* mRNA expression measured by qPCR. Data represent mean ± SD of at least three replicates; ns, not significant. (e) To detect viable bacteria inside the OTG model, the cultures were lysed at 24 h p.i. and plated for viable counting expressed as CFU/mL. Additionally, the conditioned medium (basolateral side of the insert) was plated for viable counting. Each point represents the number of CFU ± SD of at least two replicates. ***p < 0.001. (f) The localization and penetration of 33277 in gingival tissue was visualized using antibodies against bacteria (*P. gingivalis*, green; nuclei, blue; scale bar, 100 μm). (g) Green fluorescence was quantified using ImageJ software and is presented as mean ± SD. ****p < 0.0001, **p < 0.01, *p < 0.1. To confirm infection of the OTG model with bacteria, confocal microscopy was performed. (h) 3D confocal visualization was constructed from a z-stack comprising 200 images of the OTG model. Control and single- and multiple-infected cultures were costained with an anti-*P. gingivalis* antibody (green), phalloidin (actin, red), and DAPI (nuclei, blue) at 24 h p.i..; scale bar, 20 μm. Moreover, single optical slices of the OTG model showed the presence of *P. gingivalis* in epithelial cells following single and multiple inoculations. Arrows indicate intracellular bacteria. (i) Evaluation of bacterial persistence in the infected OTG model by determination of the bacterial DNA number up to 72 h p.i. Bars represent mean ± SEM. ****p < 0.0001. Similar results were obtained in at least two replicates in two independent experiments.

The OTG model was used to evaluate infection of *P. gingivalis*. For this purpose, we applied single (Figure 2a) and multiple (Figure 2b) inoculations. The latter reflects chronic exposure of gingival tissue to the pathogen. After infection with *P. gingivalis* ATCC 33277, the OTG culture was monitored for up to 72 h. Initially, using H&E staining, we analyzed the morphology of the OTG model infected with *P. gingivalis* and compared it with that of the noninfected culture (Figure 2c). Notably, we observed neither morphological changes in the structure of the epithelium (Figure 2c) nor enhanced proliferation of cells after single or multiple infections (Figure 2d), except slight shedding of the upper layer of the epithelial cells observed in multiple infections. Next, we examined the efficiency of bacterial invasion of the OTG culture and found viable bacteria in both tested models at 24 h post-infection (p.i.) (Figure 2e), with a significantly higher number of colony-forming units (CFU) identified after multiple inoculations. The infection was limited to cells that formed a 3D culture because the conditioned medium of the infected cultures on the basolateral side of the insert was sterile (Figure 2e). To examine bacterial localization and penetration in the tissue, we used specific antibodies to *P. gingivalis* (Figure 2f). Bacteria accumulated mainly in the upper layer of the epithelium in both infection models. Nevertheless, we also noticed positive staining in deep layers of the OTG culture at 24 h p.i., which indicates that some bacteria penetrated the epithelial barrier. In particular, the dissemination of *P. gingivalis* and its accumulation in deep layers were more visible in the chronic model (Figure 2f-h). Moreover, *P. gingivalis* not only disseminated through the 3D gingival epithelium (Figure 2f) but also invaded epithelial cells as bacterial cells can be observed intracellularly (Figure 2h). Persistence of bacteria was examined by determining the number of bacterial DNA copies (Figure 2i) and we found that the number of bacterial DNA copies was rapidly reduced at 24 h p.i. in the single infection model, while multiple inoculations resulted in constantly high numbers of positive readouts up to 72 h p.i. indicating for the bacterial persistence in the tissue (Figure 2i).

**Figure 3.**
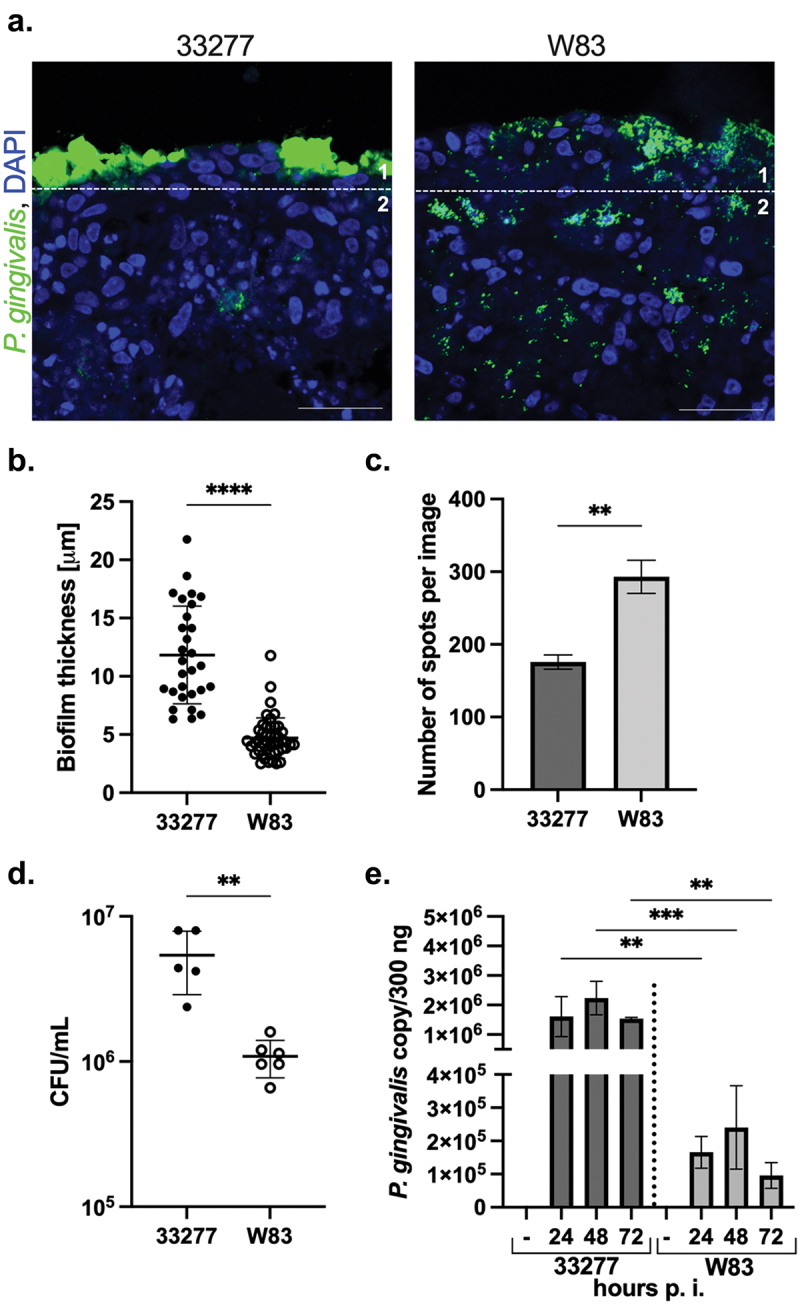
The OTG model application to differentiate the virulence potential of various *P. gingivalis* strains. (a) The localization and penetration of ATCC 33277 and W83 strains of *P. gingivalis* in gingival tissue was visualized using antibodies against bacteria (*P. gingivalis*, green; nuclei, blue; scale bar, 50 μm). (b) The thickness of biofilm was measured from sector 1; (c) The penetration of bacteria was quantified from sector 2 using ImageJ software and is presented as number of spots ± SD. ****p < 0.0001, **p < 0.01. (d) To detect viable bacteria inside the OTG model, the cultures were lysed at 24 h p.i. And plated for viable counting. The number of viable bacterial cells is expressed as CFU/mL. Each point represents the number of CFU ± SD of at least two replicates. **p < 0.01. (e) Evaluation of bacterial persistence in the infected OTG model by determination of the bacterial DNA number up to 72 h p.i. Bars represent mean ± SD. ***p < 0.001, **p < 0.01. Similar results were obtained in at least two replicates in two independent experiments.

We investigated whether the model can be applied to differentiate *P. gingivalis* strains with differing virulence characteristics using multiple infection model. We compared the W83 strain of *P. gingivalis* with ATCC 33277 (Figure 3) as they vary in the expression of fimbriae and gingipains [34,35]. *P. gingivalis* ATCC 33277 formed a thicker biofilm on the surface of OTG when compared to W83 (Figure 3a,b). On the other hand, W83 penetrated more efficiently to the deep tissue in comparison to ATCC 33277 (Figure 3c). The overall bacterial load in the OTG was higher for ATCC 33277 when measured 24 h p.i. (Figure 3d). The analysis of bacterial persistence revealed constantly higher numbers of ATCC 33277 copies up to 72 h p.i. (Figure 3e). Taken together, the data demonstrate the usefulness of the presented 3D gingival model in the investigation of infection with bacterial periopathogens differentiated in virulence.

### Monitoring the development of HSV-1 viral infection in the OTG model

**Figure 4.**
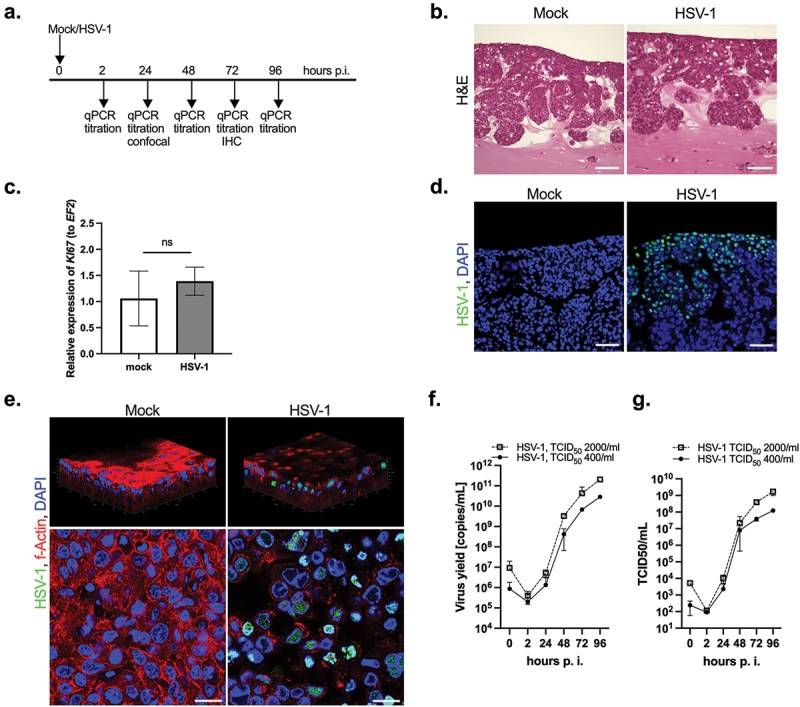
HSV-1 infection of the 3D gingival model. The OTG model was exposed to the mock or virus to allow virus adsorption and penetration. (a) Schematic representation of the experiment. (b) The morphology of the OTG model infected with HSV-1 was investigated using H&E staining; scale bar, 100 μm. (c) HSV-1 infection of the OTG model has no influence on *Ki67* mRNA expression measured by qPCR. Data represent mean ± SD of at least three replicates; ns, not significant. (d) Immunofluorescence staining with a HSV-1 antibody was performed to evaluate infection of the OTG model with the pathogen (virus, green; nuclei, blue; scale bar, 50 μm). (e) 3D confocal visualization was constructed from a z-stack comprising 200 images of the OTG model. Mock- and virus-infected cultures were costained with an anti-HSV-1 antibody (green), phalloidin (actin, red), and DAPI (nuclei, blue) at 24 h p.i.; scale bar, 20 μm. A single optical slice of the OTG model showed virus particles inside the nuclei of TIGKs. Replication kinetics of HSV-1 in the OTG model analyzed by qPCR (f) or titration (g). The OTG model was infected with two doses of the virus: TCID_50_ 400 and 2000/mL. Data points represent qPCR results or TCID_50_ of cell culture supernatants harvested at the indicated times p.i. Data are presented as HSV-1 DNA copies/mL (f) or TCID_50_/mL (g). Mean ± SD, *n* = 3.

Next, we determined whether the 3D gingival model can serve as a model for tissue infection by viral pathogens. Herpes viruses have been linked with periodontitis [9], therefore we selected HSV-1 as a model of a viral pathogen. First, we mock-infected the OTG model or treated it with HSV-1 for 2 h at 37°C to allow virus adsorption and penetration. The virus was then removed by washing the apical side of the inserts, and the progress of viral infection was monitored for 96 h (Figure 4a). Initially, evaluation of the OTG culture infected with HSV-1 by H&E staining revealed no morphological differences between the mock- and virus-inoculated epithelial layers at 72 h p.i. (Figure 4b). Notably, we observed neither signs of shedding in the apical cell layer in response to viral inoculation nor increased proliferation of cells forming the OTG model (Figure 4c). Immunofluorescence staining of the infected OTG culture visualized HSV-1-positive epithelial cells in virus-treated cultures (Figure 4d) with accumulation of virus particles in the topical layers of the gingival epithelium. Confocal imaging revealed the presence of virus particles inside the nuclei of TIGKs (Figure 4e). Analysis of the kinetics of viral multiplication by qPCR showed that after an initial lag period, HSV-1 replicated efficiently in the OTG model, with the virus yield steeply increasing at 48 h p.i. and reaching a peak of 2.05 x 10^11 (TCID_50_ 2,000/mL) or 2.85 x 10^10 (TCID_50_ 400/mL) copies/mL at 72 h p.i. (Figure 4f). Using the titration method on Vero E6 cells, we confirmed the pattern of virus replication determined by qPCR (Figure 4g).

### The effect of antibacterial and antiviral agents

3D models are a convenient tool to assess drug penetration and their efficiency against tissue invading pathogens. As we showed the persistence of pathogens in our OTG model, we tried to assess whether the model can be used to test antibacterial and antiviral drugs.

**Figure 5.**
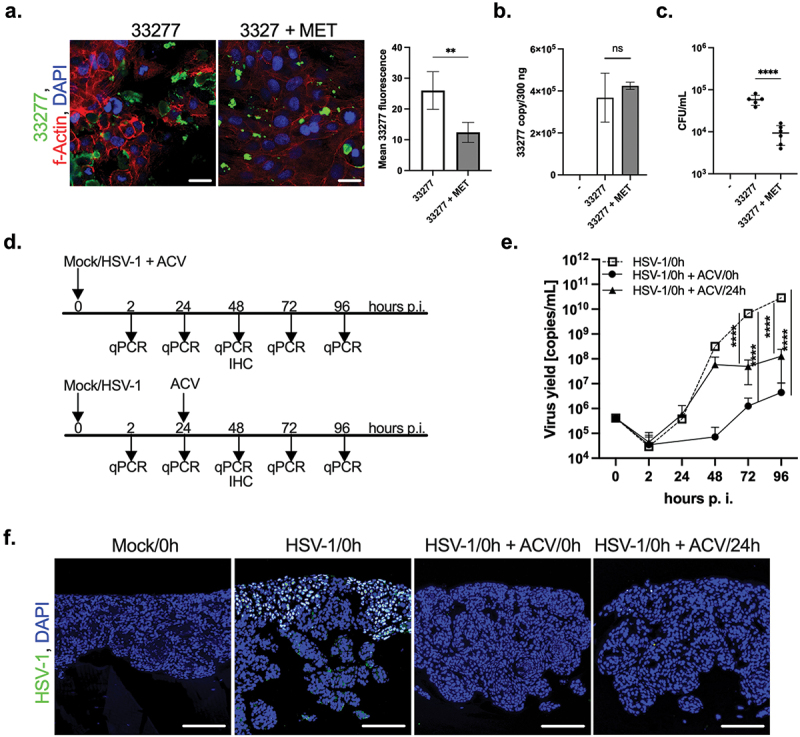
The effect of antibacterial and antiviral agents. The antimicrobial activity of metronidazole (MET) in the 33277-treated OTG model was investigated. The OTG model was infected with bacteria in the presence (33277 + MET) or absence (33277) of MET. (a) Immunofluorescence staining using antibodies to *P. gingivalis* was performed to evaluate infection of the OTG model with the pathogen (*P. gingivalis*, green; nuclei, blue; scale bar 20 μm). Green fluorescence was quantified using ImageJ software and is presented as mean ± SD. **p < 0.01. (b) The level of bacterial DNA isolated from the OTG model was determined. Bars represent mean ± SEM. ns, not significant. (c) Additionally, cells were lysed at 24 h p.i. And plated on agar plates. Each point represents the number of CFU ± SD of at least two replicates. ****p < 0.0001. The effect of ACV was administered on the day of infection (HSV-1/0 h + ACV/0 h) or 24 h p.i. (HSV-1/0 h + ACV/24 h). (d) Schematic representation of the experiment. Both treatments with ACV efficiently reduce HSV-1 infection as indicated by qPCR (e). Each point represents mean ± SD. ****p < 0.0001. The observation was confirmed by immunostaining of HSV-1 (f, virus, green; nuclei, blue; scale bar, 100 μm).

First, we studied the antibacterial activity of metronidazole in the *P. gingivalis*-infected OTG culture [36]. While metronidazole treatment caused only a limited decrease in the intensity of *P. gingivalis* immunostaining and had no effect on qPCR readouts (Figure 5a,b), the number of viable bacteria (CFU/mL) in the OTG culture exposed to metronidazole was precipitously decreased compared with the control condition (Figure 5c). Furthermore, we examined whether our OTG model can be applied to study antiviral drugs. For this purpose, we used acyclovir (ACV), which is commonly applied to treat HSV-1 infection. ACV significantly decreased herpes infection using both medication regimens (Figure 5d), as indicated by qPCR (Figure 5e) and immunostaining (Figure 5f). Notably, ACV treatment at 0 h eliminated infection more efficiently than a delayed application of the drug. This finding argues that our OTG model is suitable for analysis of the effectiveness of antimicrobial compounds.

### The host response to infection in OTG model

**Figure 6.**
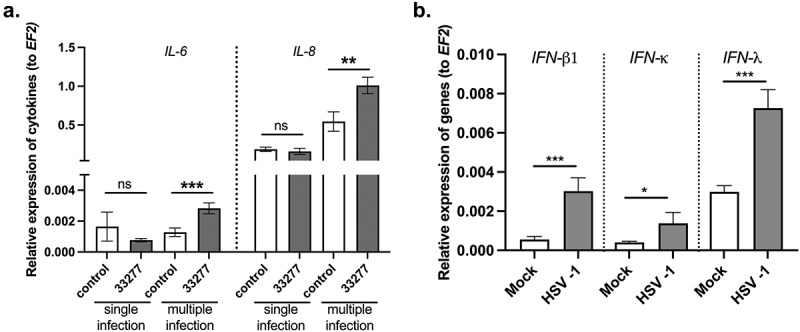
The host response to infection in OTG model. (a) Multiple infection of the OTG model with *P. gingivalis* stimulates production of *IL-6* and *IL-8* mRNA. *IL-6* and *IL-8* mRNA expression levels were measured by qPCR. Data represent mean ± SD of at least three replicates. ***p < 0.001, **p < 0.01. (b) OTG infection with HSV-1 elevates the mRNA expression levels of type I and type III interferons (IFNs). *IFN-β1*, *IFN-κ*, and *IFN-λ* mRNA expression levels were measured by qPCR. Data represent mean ± SD of at least three replicates. ***p < 0.001, *p < 0.1.

To evaluate the utility of the OTG model for study of the inflammatory response of tissue composed of gingival keratinocytes and fibroblasts, we examined the pattern of proinflammatory cytokine expression. The levels of mRNAs encoding IL-6 and IL-8 were increased in the epithelial layer of the OTG model infected with *P. gingivalis* but only upon multiple infections (Figure 6a). Moreover, to examine the immune response after viral infection, we measured the mRNA expression levels of type I and type III interferons (IFNs), which provide a first line of defense against viral pathogens. Levels of *IFN-β1*, *IFN-κ*, and *IFN-λ* transcripts were elevated compared with uninfected controls (Figure 6b). These results contend that the use of the OTG model is adequate for the study of the inflammatory response after infection with periodontal pathogens. These results also indicate that in the future the model may be expanded with an addition of immune cells.

## Discussion

The gingival epithelium constitutes the first line of defense against invading pathogens [37,38]. It is supported by an underlying layer of dense connective tissue known as the lamina propria, which consists of growth factor-secreting fibroblasts that are embedded in collagen fibers [39]. Various 3D gingival models have been developed using a variety of extracellular matrixes, epithelial cells, and fibroblasts [14–16,19,40] however, their application to the study of periodontal pathogens is under-developed. An exception is an organotypic model comprising an epithelium composed of keratinocytes derived from an oral squamous cell carcinoma of buccal mucosa keratinocytes (TR146 cells) cultured on collagen-containing primary oral fibroblasts isolated from gingival biopsies. This model was used to investigate intracellular antibiotic delivery to treat *P. gingivalis* infection [41]. Another group used a dynamic perfusion bioreactor to develop an organotypic gingival epithelial cell-fibroblast-monocyte coculture on collagen sponges [17]. Using this model, the authors investigated the interaction of host cells with bacteria, including *P. gingivalis*, grown in a biofilm. On the other hand, the application of human umbilical vein endothelial cells, which create a vascular structure in the connective tissue layer, to a 3D gingival model was recently performed to demonstrate the invasion of blood capillaries by *P. gingivalis* [20]. Another achievement in the field was the introduction of a highly advanced anatomical gingival tissue model composed of human primary cultures and resembling the human gingival pocket, which the authors proposed to be a convenient tool to study the interaction of the microbiome with the host [42].

The organotypic model presented in our study provides notable research advantages over the abovementioned models. First, it can be applied for both short- and long-term infection studies with *P. gingivalis* and enables comparison of the invasive properties of strains with different profiles of virulence factors. Second, not only adhesion but also dissemination and persistence of a pathogen can be monitored. Third, it is relatively inexpensive and easy to prepare. The commercial availability of Matrigel® as a source of extracellular matrix proteins and growth factors allows fast and efficient growth of epithelial cells on the surface. Finally, the usage of established and commercially available cell lines (TIGKs and GFs) enables easy and unlimited access to fibroblasts and epithelial cells. The application of these cell lines improves the reproducibility and consistency of the results obtained because they are donor-independent and have not been primed by any microorganisms, in contrast with primary cells obtained from healthy donors. Both cell lines grow rapidly, and their lifespans are longer than those of primary cells. Importantly, TIGKs retain the karyotype, morphology, growth, and marker protein characteristics of primary GECs [21]. These immortalized GECs accurately mimic the responses of primary GECs and thus are a valuable tool for studying the interactions of subgingival pathogens with epithelial cells such as those encountered in vivo [21]. The limitation of GF cell line usage is the lack of the TLR-2 receptor, which can be overpassed by the application of genetically modified fibroblasts [43].

Regardless of a model a general drawback of studying interactions of anaerobic bacteria with eukaryotic cells is requirement of the oxygen presence during the co-culture, which may have deleterious effect on anaerobe vitality. Keeping this in mind we checked *P. gingivalis* liveliness by plating and CFU counting and shown the presence of live bacteria 24 hours p.i. in infected OTG. Presumably, the rapid penetration of bacteria into the tissue, where the partial oxygen pressure is reduced allows the survival. This finding corroborates with intracellular viability of *P. gingivalis* under aerobic growth conditions during infection of organotypic oral mucosa model [44]. Nevertheless, it is important to evaluate the oxygen tolerance of anaerobic pathogens before examination the interaction between pathogens and 3D organotypic cultures. Another issue worth considering is the presence of immune cells, osteoblasts, or endothelial cells. The enrichment of OTG with those cells would more closely resemble the cellular composition of the periodontal tissue. Such model will be a very valuable tool to investigate bacterial and viral infections in the pathogenesis of periodontitis.

Considering the increase in clinical data showing the impact of HSV-1 infection on periodontitis development [9,45,46], our organotypic model can be used as a robust tool to study bacterial and/or viral infections underlying the pathobiology of periodontitis. Our report is the first to describe herpes simplex virus type 1 infection in a 3D gingival model. We monitored adhesion of the virus, its penetration into deep layers of the tissue, and, most importantly, its reproduction. Until now, the biology of HSV-1 has been studied using mouse fibroblasts encapsulated in a hydrogel composed of acrylated hyaluronic acid [47], a human keratinocyte cell line (HaCaT) on a collagen substrate containing human primary fibroblasts [48], or brain organoids made from human induced pluripotent cells [49]. Growing evidence implicates HSV-1 in the development of periodontitis, and our 3D gingival model will be useful for studying periodontal herpes infection.

In summary, the presented OTG model constitutes simple and convenient tool to study host-pathogen interactions in the context of molecular mechanisms underlying the pathogenesis of periodontitis. It allows examination of the host’s response to infection with various types of oral pathogens, including bacteria and viruses. Finally, it is also suitable to investigate the efficiency of therapeutics for periodontal diseases.
